# Supplementary material for: Immune transcriptomes of highly exposed SARSCoV-2 asymptomatic seropositive versus seronegative individuals from the Ischgl community
Source: Res Sq. 2020 Sep 23:rs.3.rs-69657. Preprint. [Version 1] doi: 10.21203/rs.3.rs-69657/v1 (PMC7523134; doi:10.21203/rs.3.rs-69657/v1)
Supplement: Supplement [file Supplementaryinformation.docx]

**Supplementary Information**

**Immune transcriptomes of highly exposed SARS-CoV-2 asymptomatic seropositive versus seronegative individuals from the Ischgl community**

Hye Kyung Lee^1, *^, Ludwig Knabl^2,*,†^, Lisa Pipperger^2^, Andre Volland^2^, Priscilla A. Furth^3^, Keunsoo Kang^4^, Ludwig Knabl Sr.^5^, Romuald Bellmann^6^, Christina Bernhard^7^, Norbert Kaiser^8^, Hannes Gänzer^9^, Mathias Ströhle^10^, Andreas Walser^11^, Dorothee von Laer^2^ and Lothar Hennighausen^1,†^

^*^ Equal contribution

^†^ Correspondence to: L.K. ([Ludwig.knabl@i-med.ac.at](mailto:Ludwig.knabl@i-med.ac.at)) and L.H. ([lotharh@niddk.nih.gov](mailto:lotharh@niddk.nih.gov))

Supplementary Table 1. Household of tested patients.

Supplementary Table 2. A list of genes and GSEA analysis for mild symptomatic and seronegative patients of non-lschgl residents (Group D and E).

Supplementary Table 3. Gene list and GSEA analysis for asymptomatic and seronegative patients (Group A and B).

Supplementary Table 4. Cytokine profiles of all individuals.

Supplementary Table 5. Gene expression profiles and GSEA analysis from the cystic fibrosis patient and asymptomatic patients (Group A).

Supplementary Table 6. Gene expression profiles and GSEA analysis from the NEMO deficient patient and asymptomatic patients (Group A).
